# Effort–reward imbalance and burnout among German school leaders. Representative study findings and implications for an underserved occupational group

**DOI:** 10.1093/eurpub/ckae182

**Published:** 2024-11-28

**Authors:** Nele Groß, Kevin Dadaczynski, Marcus Pietsch

**Affiliations:** Institute of Educational Sciences, Leuphana University Lueneburg, Lueneburg, Germany; Health Sciences, Fulda University of Applied Sciences, Fulda, Germany; Institute of Educational Sciences, Leuphana University Lueneburg, Lueneburg, Germany

## Abstract

This study examines the relationship between gratification crisis and burnout syndrome in German school leaders and explores how sociodemographic and school-level factors relate to burnout. Additionally, it investigates how these factors influence the likelihood of experiencing burnout and engagement based on sociodemographic and school characteristics. The research design focuses on the relationship between the effort–reward imbalance (ERI) model and latent profiles of the Maslach Burnout Inventory. Statistical analyses include group comparisons and logistic regression to examine the relationships between variables, such as ERI and sociodemographic and school-level characteristics. The ERI ratio significantly associated with burnout, indicating higher risk as the ratio rises. Sociodemographic and school-level factors showed significant variations by student numbers and school type. Regression models revealed a positive relationship between ERI and burnout, while age negatively correlated, suggesting lower burnout risk in older leaders. Logistic regression highlighted associations between age, gender, and engagement, with increasing age positively associated with engagement and lower ERI scores associated with higher engagement. The results of the study demonstrate a significant relationship between perceived reward crisis as measured by the ERI model and burnout syndrome among school leaders in Germany. School leaders who feel undervalued and overburdened are more susceptible to burnout. The reported ERI depends on specific contextual variables, including the type of school, the number of students, and the age of the school principal. It is important for schools and educational institutions to address the multiple factors that influence burnout and work engagement among school leaders.

## Introduction

In addition to physical health, mental health is a key component of a comprehensive health concept of health [1]. Recent evidence suggests that mental stress at work is a significant public health issue, leading to considerable societal costs [2, 3]. Mental illnesses cause individual suffering and negatively impact work performance. Negative job-related factors, like heavy workloads, time pressure, and perceived unfair pay, increase psychosocial stress and the risk of adverse mental health outcomes [4], reducing work ability and increasing sick leave and early retirement [5].

A high-quality school, defined by effective teaching and a stimulating environment, depends heavily on educators’ well-being. School leaders are positioned to competently fulfill their leadership roles only if they maintain sufficient physical and mental health. Resulting from the Global Educational Reform Movement, the roles and duties of school principals have undergone substantial changes in recent years. Although schools and their leaders have gained more autonomy, influences at the system level have also increased (e.g. national standardized tests and curricula, national school inspections). School principals are involved in a wide range of activities, that range from administrative and management tasks, communication, and collaboration to financial management, infrastructure improvements, and implementation of educational policies. In a German study, Dadaczynski *et al*. [6] found that more than a quarter of school principals reported poor general health, and more than one-third were dissatisfied or very dissatisfied with their work.

According to Julmi and Scherm [7], burnout develops when a person faces emotionally uncontrollable, stressful situations that overwhelm their resources and capacities. Polndorfer [8] defines it as exhaustion resulting from internal or external excessive demands. Burnout syndromes can negatively impact work performance, satisfaction, and motivation. School leaders handle a wide range of tasks, including administration, management, communication, collaboration, financial management, infrastructure improvements, and implementation of educational policies. Consequently, they often juggle school effectiveness and improvement, frequently switching between tasks [9, 10], which can lead to cognitive strain [11] and stress [12].

The effort–reward imbalance (ERI) model, or Siegrist’s model of occupational gratification crises, helps to explain the relationship between work-related psychosocial stress and health and has proven to be a useful tool for examining work-related mental health. Professional effort is considered part of a social contract; in return, there is a reward of salary, recognition, or job security, known as social reciprocity [13]. According to the ERI model, work that involves high effort but low satisfaction leads to a disruption in the balance between costs and benefits [14]. If there is a lack of reciprocity, in the form of high costs and low returns, strong negative feelings and stress reactions can arise, which then lead to the activation of the autonomic nervous system [15]. If there is an imbalance between effort and the reward at work, this can lead to a crisis of job satisfaction, known as gratification crisis, which increases the risk of stress and mental illness [16]. Research has shown that work stress, as measured by the ERI, is a good predictor of mental illnesses such as depression [17].

The ERI model has already been applied in various studies within the school context, particularly concerning the mental health of teachers and educational staff. There is no evidence regarding ERI and the mental health of teachers [18, 19]. Backhaus *et al*. [20] examined the association between the disparity between effort and reward and its link to burnout and somatic symptoms in German kindergarten educators. Furthermore, Unterbrink *et al*. [21] found high rates of burnout symptoms, such as emotional exhaustion, depersonalization, and low personal performance in teachers from Germany.

Based on the available evidence-base, this study aims to examine the association between school leaders’ gratification crisis and symptoms of burnout. Additionally, as shown above, sociodemographic and school-level characteristics are considered in order to better understand these associations. The following research questions guided this study:

- To what extent are German school leaders at risk of experiencing a gratification crisis?
- Are there differences between sociodemographic and school-level characteristics in terms of effort, reward, and the ERI?
- To what extent does the likelihood of experiencing burnout and engagement vary according to sociodemographic and school-level characteristics?

## Methods

### Study design and participants

We reanalyzed data from the first wave of the longitudinal study Leadership in German Schools (LineS) [22], conducted in the fall of 2019. In Germany, education is the responsibility of the 16 federal states, leading to variations in the school system. Primary education (*Grundschulen*) is standardized nationwide for children aged 6–10. At around 11, students enter the fifth grade of secondary school, where they are divided into different types of schools (secondary general school [*Hauptschule*], intermediate school [*Realschule*], and grammar school [*Gymnasium*]) depending on their abilities and teachers’ recommendations. Comprehensive schools have been introduced in many states as part of educational reforms; these are a type of school at the lower secondary level offering several courses of education leading to different qualifications. Each school usually has a school principal who has been a teacher, although qualifications and professional requirements vary widely from state to state [23].

In each wave of the Leadership in German Schools study, a random sample of school leaders (principals and deputy principals) was surveyed by the field service provider Forsa Institute for Social Research and Statistical Analysis. The study was conducted with informed consent and in accordance with the applicable rights under the general data protection regulation. Participants were informed that participation was voluntary and that their anonymized data would be protected in accordance with the legal requirements.

School leaders were identified on a random basis, leading to a nationally representative sample for general schools in Germany. A total of 650 school leaders identified using this method were invited to participate in an online survey, also administered by Forsa (participating respondents, *N* = 405, response rate 62%). Deviations of the sample from the population were adjusted *post hoc*. For this purpose, weights were created in an iterative process using the structuring variables. Accordingly, there was a corresponding (individual) weight for each case in the dataset.

### Measures

In this study, the ERI score served as an independent variable, with symptoms of burnout being the dependent variable. Sociodemographic and school-level characteristics, such as age, type of school, geographic location, number of students, and weekly working hours, were also included.

To examine the ERI quotient, three items from the effort domain and four items from the reward domain were selected from the short version of the ERI instrument developed by Siegrist *et al*. [13] In Germany, school leaders are usually civil servants with tenure, so questions about questions about professional future (ERI 5 and ERI 7) and change in work situation (ERI 6) were not used in the study. The adaption of the number of items was considered with the correction factor when calculating the ERI ratio. The items were answered in two steps. First, school leaders agreed or disagreed with the item content describing a typical experience of their work situation. Then, those who agreed were asked to rate the extent to which they were affected by this typical experience using a four-point Likert scale.

For reliability analysis, Cronbach’s alpha was calculated to assess the internal consistency of the subscales. The Cronbach’s alpha measure of reliability was acceptable for the whole effort scale: α = 0.79. The reward scale had a value of α = 0.75. According to Loewenthal [24], Cronbach’s alpha coefficients of α > 0.60 can be considered acceptable for scales with less than 10 items.

For this study, which pursues an economic screening approach, one item of the three dimensions of the Maslach Burnout Inventory (MBI) [25] was selected:

1. Dimension *depersonalization/cynicism*: I have become more callous toward people since I took this job as a school principal,
2. Dimension *personal accomplishment*: I have accomplished many worthwhile things in my current job,
3. Dimension *emotional exhaustion*: I feel fatigued when I get up in the morning and have to face another day on the job.

The items were rated on a four-point response format ranging from one (do not agree at all) to four (agree fully). Item 2 was recoded for analyses.

The three dimensions of burnout do not always progress simultaneously, indicating that they are not highly correlated enough to be considered a single, one-dimensional phenomenon. The advantage of having these distinct yet interrelated dimensions of burnout is that individuals may exhibit different patterns at different times [26]. A person-centered approach, like profile analysis, is essential for understanding all dimensions of burnout. Unlike quantitative comparisons, this method focuses on identifying qualitative differences between individuals. It emphasizes that deviations from typical patterns can have significant consequences.

### Sociodemographic and school-level characteristics

Biological gender was assessed using three response options (male, female, diverse), while age was assessed as open information and categorized subsequently into age groups (1 = 30–44 years; 2 = 45–59 years; 3 = more than 60 years). Various indicators were examined to characterize the schools. In addition to type of school, geographical location was examined (1 = village, less than 3000 inhabitants; 2 = town, 3000–15 000 inhabitants; 3 = small town, 15 000–100 000 inhabitants; 4 = larger city, 100 000–1 000 000 inhabitants; and 5 = metropolis, more than 1 000 000). To determine the school’s size, school leaders were asked about the approximate number of students (1 = 1–100; 2 = 101–200; 3 = 201–300; 4 = 301–500; 5 = 501–1000; 6 = more than 1000). The working hours per week were assessed in order to determine the workload (1 = less than 35 h; 2 = 3–40 h; 3 = 41–50 h; 4 = more than 50 h).

### Statistical analyses

Descriptive statistics and preparation of data for multivariate analyses were carried out using IBM SPSS 28 (New York) software [27]. The multivariate analyses were done in MPlus 8.3 (Los Angeles) software [28] to avoid bias due to missing estimators of the true value.

The ERI-related health risk results were estimated from the calculated relationship between effort and reward (ERI ratio), with attention given to the weighting factor by using a factor that balances the unequal number of elements of the effort and reward ratings. To analyze group differences, an analysis of variance (ANOVA) was conducted, considering the various sociodemographic and school-level variables. When equality of variances could be assumed and slightly different numbers of cases were available, the Gabriel *post hoc* test according to Field [29] was used.

To validate Leiter and Maslach’s [26] latent profile analysis, latent class analysis (LCA) was used to check the extent to which different types could be analyzed. The results of the analyses indicated the presence of two classes (Akaike’s Information Criterion (AIC) = 2043.420/Bayesian Information Criterion (BIC) = 2119.400/adjusted BIC = 2059.111). The Voung-Lo-Mendell-Rubin (VLMR) test [30] also showed that the two-class model fit significantly better than the one-class model (*P* = .0013) and the three-class model (*P* = .8390).

The first class comprises 38.6% of the cases and the second class 61.3% of the cases. The average for the latent class probabilities for most likely latent class membership (row) by latent class (column) are 0.749 for class 1 and 0.876 for class 2. People in group 1 score low on depersonalization/cynicism (*n* = 171, *M* = 1.58; SD = 0.71), but higher on personal accomplishment (*n* = 171, *M* = 2.02; SD = 0.49) and emotional exhaustion (*n* = 171, *M* = 2.27; SD = 0.73). People in group 2 also score low on depersonalization/cynicism item (*n *=* *229, *M *=* *1.11; SD* *=* *0.40), higher on personal accomplishment item (*n *=* *220, *M *=* *1.49; SD* *=* *0.53), but lower on the emotional exhaustion item (*n *=* *220, *M *=* *1.16; SD* *=* *0.43).

The two dichotomous variables BURNOUT and ENGAGED were created for the logistic regression, and these were analyzed using a person-oriented approach and a latent profile, according to Leiter and Maslach [26]. To determine odds ratios (OR), both BURNOUT and ENGAGED were formed as particular profile classifications. BURNOUT was captured by three subscales: high exhaustion, high depersonalization/cynicism, and low personal accomplishment. In contrast, ENGAGED was associated with low exhaustion, low depersonalization/cynicism, and high personal accomplishment [26, 31].

## Results

A total of 405 school leaders (principals and deputy principals) from schools in Germany were included in the final analysis (see Table 1). Of the schools that were administered, 91.8% were state schools and 8.2% were private schools. Just above 15% of respondents indicated that their school was located in a “social hotspot,” or an area characterized by high poverty and unemployment rates. Over half (53.5%) of the respondents reported working between 41 and 51 h in an average school week (including overtime), and over a quarter (27.4%) reported working more than 50 h.

**Table 1.** Sample characteristics (absolute and relative frequencies; weighted)

	Frequencies		
	*n*		%
Gender (*n *=* *405)			
Male	183		45.3
Female	220		55.8
Diverse	1		0.4
Age (*n *=* *405)			
30–44 years	46		11.4
45–59 years	230		56.9
>60 years	128		31.7
Type of school (*n *=* *405)			
Primary schools	202		49.9
Secondary general schools (*Hauptschule*)	28		6.8
Middle secondary schools (*Realschule*)	24		6.0
Grammar schools (*Gymnasium*)	41		10.1
Comprehensive schools	28		6.9
Schools with several educational programs (e.g. district schools or community schools)	24		6.0
Schools for children with special educational needs	37		9.2
Other types of school	21		5.1

The descriptive analysis showed that the efforts are more often reported than the rewards (see Table 2). Considering both ERI dimensions separately, neither effort nor reward show any associations with burnout in either the regression or the model.

**Table 2.** Overview of the agreement of the participants who agreed with the ERI statements in the first step

				Frequencies in percentages			
Items	*n*	*M*	SD	Not at all	Moderate	Strong	Very strong
Effort							
I have constant time pressure due to a heavy workload.	362	2.8	0.70	0.5	35.1	48.3	16.1
I have many interruptions and disturbances while performing my job.	376	2.71	0.79	3.8	38.4	40.6	17.2
Over the past few years, my job has become more and more demanding.	352	2.93	0.75	1.3	27.3	48.4	22.8
Reward							
I receive the respect I deserve from my superior or a respective relevant person.	260	3.01	0.76	0.6	26.7	44.1	28.6
Considering all my efforts and achievements, I receive the respect and prestige I deserve at work.	230	2.37	0.75	7.6	56.8	26.6	9.0
Considering all my efforts and achievements, my job promotion prospects are adequate.	109	2.16	0.76	15.4	59.6	18.8	6.3
Considering all my efforts and achievements, my salary/income is adequate.	235	2.34	0.81	11.5	53.0	25.3	10.2

*M,* mean; *n*, sample; SD, standard deviation.

With regards to research question 1, the findings show that approximately one quarter (24%) of the respondents experience more reward than effort and are not at risk of a gratification crisis. However, 73.9% of the respondents who perceived more effort than reward were exposed to a gratification crisis. For 1.3% of participants, effort and reward were in balance.

With regard to research question 2, we found no gender differences for ERI. In addition, no ERI differences could be found for age, or the geographical location of the school (village, town, small town, larger city, metropolis).

In turn, significant ERI differences could be observed with regard to the number of students enrolled at school, with school leaders from schools with higher numbers of students (501–1000) showing a higher frequency of experiencing a gratification crisis (*F*(6, 39) = 2.8, *P* < .05). With regard to the type of school, school leaders from primary schools and those from secondary general school (*Hauptschule*) had a higher risk of experiencing a gratification crisis than school leaders from schools for students with special needs (*F*(7, 39) = 2.04, *P* < .001). For the reward dimension, significant types of school differences could be found (*F*(7, 39) = 3.66, *P* < .001). Respondents from schools for children with special education needs reported a higher reward in their work compared to respondents from primary and secondary general schools (*Hauptschule*).

Using the sociodemographic and school characteristics presented, a logistic regression model was created for the latent class BURNOUT (see Table 3). With regard to research question 3, the extension of the findings shows different results with regard to the other independent variables in the model. Nonetheless, for age, a significant negative correlation could be observed. Older participants exhibited a decrease in burnout risk. In addition, the analyses of the ERI score show a statistically significant correlation.

**Table 3.** Regression coefficients and *P*-values for the predictors of BURNOUT

*N* = 405	Model 6		Model 5		Model 4		Model 3		Model 2		Model 1	
	*b*	*P*-value	*b*	*P*-value	*b*	*P*-value	*b*	*P*-value	*b*	*P*-value	*b*	*P*-value
Secondary schools^a^	0.03	.77	0.14	.047*	0.126	.196	−0.03	.807	−0.03	.735	−0.22	.003*
Other types of schools^a^	0.058	.064	0.14	.047*	0.126	.196	−0.03	.807	−0.03	.735	−0.22	.003*
ERI score			0.14	.045*	0.14	.14	0.00	.983	−0.04	.599	−0.23	.001*
Geographical location^b^					0.13	.183	−0.01	.953	−0.04	.484	−0.23	.002*
Number of students							0.08	.315	−0.03	.732	−0.23	.001*
Hours of work per week									0.02	.830	−0.22	.003*
Age											−0.23	.002*

*b*, standardized regression coefficients.

a Reference category: elementary schools.

b Reference category: village.

*P <* *.05.

A dichotomous calculation of BURNOUT showed that 15.8% (*n* = 63) of the respondents are at risk of burnout, while 83.2% (*n* = 337) are currently not at risk of burnout in a latent profile analysis. However, the latent profile analysis regarding ENGAGED showed that only 19.4% (*n* = 75) of school leaders are engaged in their work. Logistic regression analyses were performed to examine the associations between the independent variables and the dichotomous outcome variables (BURNOUT and ENGAGED). The results of the OR are summarized in Table 4.

**Table 4.** Results of the logistic regression for the latent profiles BURNOUT and ENGAGED (*N* = 405)

Independent variable	OR	95% CI	SE	*P*-value
Burnout on				
Demographic factors				
Age	0.501	0.32–0.79	0.116	<.001*
Gender^a^	0.652	0.36–1.20	0.203	.087^+^
School-level factors				
Secondary schools^b^	1.228	0.61–2.46	0.434	.600
Other types of schools^b^	1.980	0.53–7.34	1.323	.459
Number of students	1.001	0.78–1.29	0.128	.993
Hours of work per week	0.922	0.68–1.25	0.142	.583
Geographic location^c^	1.963	0.77–4.99	0.934	.303
ERI score	1.105	1.00–1.22	0.054	.053^+^
Engaged on				
Demographic factors				
Age	2.874	1.83–4.52	0.662	.005*
Gender^a^	0.549	0.31–0.97	0.160	.005*
School-level factors				
Secondary schools^b^	0.912	0.48–1.73	0.289	.767
Other types of schools^b^	0.483	0.06–4.05	0.524	.323
Number of students	0.995	0.78–1.27	0.123	.965
Hours of work per week	0.923	0.70–1.23	0.133	.566
Geographic location^c^	0.959	0.44–2.15	0.393	.938
ERI score	0.882	0.80–0.97	0.043	.006*

a Reference category: male.

b Reference category: elementary schools.

c Reference category: village.

*P <* *.05.

+ *P*_one-tailed_ < .05.

With respect to research question 4, the findings indicate that a unit increase in age (1 year of life) increases the odds of ENGAGED by a factor of 2.87. Being female increases the odds of ENGAGED by a factor of 0.549. Conversely, a lower ERI score is significantly associated with an increase in ENGAGED (OR = 0.88, 95% CI = 0.80–0.97, *P *<* *.05). This means that for every unit increase in the independent variable ERI score (i.e. less perceived reward than effort), the probability of ENGAGED decreases by 12%. For all remaining independent variables no significant associations with the outcome variables BURNOUT and ENGAGED could be found.

## Discussion

Although school health promotion is high on the agenda of Public Health policy and research worldwide, the health of school principals has long been neglected. Based on the results, the research questions can be answered as follows:

*To what extend are German school leaders at risk of experiencing a gratification crisis?* The study shows that a significant number of school leaders in Germany are under severe time pressure, indicating a high workload. In addition, a significant number of them have the feeling that they are not adequately rewarded for their work. The high workload and the imbalance between effort and reward are crucial factors contributing to the psychological stress of school leaders.

*Are there differences between sociodemographic and school-level characteristics in terms of effort, reward, and the ERI?* The results suggest a strong relationship between reward crises, as measured by the ERI model and burnout syndrome. An elevated ERI ratio, indicating a greater perception of effort relative to reward, is associated with a higher risk of burnout. This suggests that school leaders who feel undervalued and overworked are more prone to burnout. The ERI model can therefore serve as a useful tool for identifying the risk of burnout among school leaders and for taking preventive action.

*To what extent does the likelihood of experiencing burnout and engagement vary according to sociodemographic and school-based characteristics?* In summary, the logistic regression has yielded valuable insight into the correlation between age, burnout, and engagement. The observed OR for age in relation to burnout implies a protective effect, suggesting that with each year that passes, the likelihood of experiencing burnout decreases. Conversely, the positive association between age and engagement suggests that the likelihood of engagement increases significantly with age. Age acts as a predictor for a positive progression of mental health in school principals. Various explanations may exist for the increased risk of burnout syndrome among younger principals: as they age, they generally gain more experience and proven coping strategies to deal with stress and the demands of running a school [32]. Older school principals may place more value on their health, family, and leisure time and incorporate this into their decisions and work practices to prevent burnout [33]. They also tend to have stronger social and professional networks, which help manage work pressures and emotional stress [34]. Similarly, priorities may change with age, leading to a more balanced work–life experience. Older school leaders may place more value on their health, family, and leisure time and incorporate this into their decisions and work practices to prevent burnout [35]. However, these benefits of age vary among individuals and are not universal. To ensure the long-term mental health of school principals, identifying and implementing reward measures is essential.

While the present study provides valuable insights into the associations between ERI, burnout, and engagement in school principals, several limitations must be considered. Although quantitative methods are suitable for analyzing large amounts of data, comprehensive qualitative research is necessary to thoroughly investigate the specific factors that contribute to work-related stress and lack of recognition among school principals. This will provide a thorough understanding of their experiences. Comparative research studies evaluate how differentiating factors, such as school types, geographical locations, and other relevant variables, affect the psychological well-being of school principals. Furthermore, the study’s cross-sectional design prohibits the establishment of causal relationships, and the reliance on self-reported data introduces the possibility of response bias.

In practice, the results of the data analysis show that the development of evidence-informed intervention programs should focus on current results. The results should lead to a reduction in identified stressors and a crisis in gratification through rewards that are perceived as appropriate. The implementation of training programs should focus on enhancing the leadership skills, resilience, and adaptability of school administrators to effectively manage the numerous challenges they face in their role. School principals are often expected to be selfless and to put the needs of others before their own. Such norms, combined with a complex organizational environment with high demands and limited resources, may contribute to stress and burnout, as our findings confirm. It is therefore important to emphasize that future intervention strategies should go beyond individual-level measures to address work-related stress by also creating health-promoting working conditions that support a balance between effort and reward.

The psychological stress of school leaders in Germany is a complex challenge influenced by various factors and working conditions. The study shows a significant link between perceived reward crisis, measured by the ERI model, and burnout among school principals. Principals who feel undervalued and overburdened are more susceptible to burnout and mental health issues. This ERI is influenced by contextual factors such as the type of school, number of students, and principal’s age. Understanding these factors is crucial for developing measures to prevent mental illness and promote mental health.

The study highlights the complexity of burnout development among school principals. Contextual conditions affect ERI, which in turn affects burnout. In addition to models that allow the study of indirect associations [36], machine learning approaches can be used to analyze large amounts of data and nonlinear relationships [37]. Given the paucity of evidence on the health and well-being of school principals from non-Anglophone countries [38], we also recommend that such studies be conducted regularly in other countries. A screening approach such as the one presented here could be a cost-effective option that can be implemented quickly.

Conflict of interest: None declared.

## Data Availability

Requests to access the dataset should be directed to M.P. (marcus.pietsch@leuphana.de). Key points Significant link found between perceived reward crisis (ERI model) and burnout among school leaders. School leaders feeling undervalued and overburdened are more prone to burnout and mental health issues. ERI is influenced by school type, student number, and school leader’s age. Understanding these factors is crucial for preventing mental illness and supporting school leaders’ mental health. Findings can guide interventions to improve working conditions for school leaders.

## References

[1] World Health Organization. Mental Health: Facing the Challenges, Building Solutions: Report from the WHO European Ministerial Conference. Copenhagen: WHO Regional Office for Europe, 2005.

[2] Hassard J , Teoh KRH, Visockaite G et al The cost of work-related stress to society: a systematic review. J Occup Health Psychol 2018;23:1–17. 28358567 10.1037/ocp0000069

[3] Schütte M , Windel A. Psychische Gesundheit in der Arbeitswelt—Wissenschaftliche Standortbestimmung. Z Arb Wiss 2017;71:1–5.

[4] Siegrist J. Die ärztliche Rolle im Wandel. Bundesgesundheitsblatt Gesundheitsforschung Gesundheitsschutz 2012;55:1100–5. 22936476 10.1007/s00103-012-1527-y

[5] Rajgopal T. Mental well-being at the workplace. Indian J Occup Environ Med 2010;14:63–5. 21461156 10.4103/0019-5278.75691 PMC3062016

[6] Dadaczynski K , Okan O, Messer M. Belastungen Und Beanspruchungen von Schulleitungen Während Der Corona-Pandemie: Ergebnisse Einer Online-Befragung in Vier Bundesländern. Fulda, Bielefeld, Trier: Public Health Zentrum Fulda (PHZF) an der Hochschule Fulda, Interdisziplinäres Zentrum für Gesundheitskompetenzforschung (IZGK) an der Universität Bielefeld & Pflegewissenschaft II an der Universität Trier, 2021.

[7] Julmi C , Scherm E. Burnout trotz geringer Anforderungen: Warum auch Arbeitslose an Burnout erkranken können. SEM Radar 2013;12:17–27.

[8] Polndorfer C. Burnout. In: Hallenberger F, Lorei C (eds), Grundwissen Stress. Frankfurt: Verlag für Polizeiwissenschaft, 2014, 323–55.

[9] Dedering K , Pietsch M. School leader trust and collective teacher innovativeness: on individual and organisational ambidexterity’s mediating role. Educ Rev 2023;1:1–30.

[10] Pietsch M , Tulowitzki P, Cramer C. Innovating teaching and instruction in turbulent times: the dynamics of principals’ exploration and exploitation activities. J Educ Change 2023;24:549–81. 10.1007/s10833-022-09458-2 PMC9127291 40479231

[11] Keller T , Weibler J. What it takes and costs to be an ambidextrous manager: linking leadership and cognitive strain to balancing exploration and exploitation. J Leadersh Organ Stud 2015;22:54–71.

[12] Hunter ST , Cushenbery LD, Jayne B. Why dual leaders will drive innovation: resolving the exploration and exploitation dilemma with a conservation of resources solution. J Organiz Behav 2017;38:1183–95.

[13] Siegrist J , Starke D, Chandola T et al The measurement of effort–reward imbalance at work: European comparisons. Soc Sci Med 2004;58:1483–99. 14759692 10.1016/S0277-9536(03)00351-4

[14] van Vegchel N , de Jonge J, Bosma H et al Reviewing the effort-reward imbalance model: drawing up the balance of 45 empirical studies. Soc Sci Med 2005;60:1117–31. 15589679 10.1016/j.socscimed.2004.06.043

[15] Siegrist J. Effort-reward imbalance at work and cardiovascular diseases. Int J Occup Med Environ Health 2010;23:279–85. 20934954 10.2478/v10001-010-0013-8

[16] Siegrist J. Adverse health effects of high-effort/low-reward conditions. J Occup Health Psychol 1996;1:27–41. 9547031 10.1037//1076-8998.1.1.27

[17] Siegrist J , Dragano N. Psychosoziale Belastungen und Erkrankungsrisiken im Erwerbsleben. Befunde aus internationalen Studien zum Anforderungs-Kontroll-Modell und zum Modell beruflicher Gratifikationskrisen. Bundesgesundheitsbl 2008;51:305–12. 10.1007/s00103-008-0461-5 18369565

[18] Gluschkoff K , Elovainio M, Keltikangas-Järvinen L et al Stressful psychosocial work environment, poor sleep, and depressive symptoms among primary school teachers. Electron J Res Educ Psychol 2017;14:462–81.

[19] Hinz A , Zenger M, Brähler E et al Effort-reward imbalance and mental health problems in 1074 German teachers, compared with those in the general population: effort-reward imbalance in teachers. Stress Health 2016;32:224–30. 25053122 10.1002/smi.2596

[20] Backhaus O , Hampel P, Dadaczynski K. Effort-reward imbalance and burnout in German kindergarten educators. Eur J Health Psychol 2018;25:73–82.

[21] Unterbrink T , Hack A, Pfeifer R et al Burnout and effort–reward-imbalance in a sample of 949 German teachers. Int Arch Occup Environ Health 2007;80:433–41. 17294239 10.1007/s00420-007-0169-0

[22] Cramer C , Ophoff JG, Pietsch M et al Schulleitung in Deutschland. Repräsentative Befunde zur Attraktivität, zu Karrieremotiven und zu Arbeitsplatzwechselabsichten. Die Deutsche Schule 2021;2021:132–48.

[23] Tulowitzki P , Hinzen I, Roller M. Die Qualifizierung von Schulleiterinnen in Deutschland—ein bundesweiter Überblick. Die Deutsche Schule 2019;111:149–69.

[24] Loewenthal K. An Introduction to Psychological Tests and Scales. New York: Garland Science, 2020.

[25] Maslach C , Jackson SE, Leiter MP. Maslach Burnout Inventory Manual. Palo Alto, CA: Consulting Psychologists Press, 1996.

[26] Leiter MP , Maslach C. Latent burnout profiles: a new approach to understanding the burnout experience. Burn Res 2016;3:89–100.

[27] IBM Corp. IBM SPSS Statistics for Windows, Version 28.0. Armonk, NY: IBM Corp., 2021.

[28] Muthén LK , Muthén BO. Mplus, Version 8.3. Los Angeles, CA: Muthén & Muthén, 2019.

[29] Field A. Discovering Statistics Using IBM SPSS Statistics. 4th edn. Los Angeles, London, New Delhi: SAGE Publications; 2013.

[30] Lo Y. Testing the number of components in a normal mixture. Biometrika 2001;88:767–78.

[31] Mindgarden.com. *A message from the Maslach Burnout Inventory Authors.* https://www.mindgarden.com/blog/post/44-a-message-from-the-maslach-burnout-inventory-authors (16 August 2024, date last accessed).

[32] Hargreaves A. Educational change takes ages: life, career and generational factors in teachers’ emotional responses to educational change. Teach Teach Educ 2005;21:967–83.

[33] Betschart S , Sandmeier A, Skedsmo G et al The importance of school leaders’ attitudes and health literacy to the implementation of a health-promoting schools approach. Int J Environ Res Public Health 2022;19:14829. 36429547 10.3390/ijerph192214829 PMC9690102

[34] Oplatka I. Principals in late career: toward a conceptualization of principals’ tasks and experiences in the pre-retirement period. Educ Adm Q 2010;46:776–815.

[35] Oplatka I. Towards a conceptualization of the early career stage of principalship: current research, idiosyncrasies and future directions. Int J Lead. Educ 2012;15:129–51.

[36] Hayes AF , Preacher KJ. Statistical mediation analysis with a multicategorical independent variable. Br J Math Stat Psychol 2014;67:451–70. 24188158 10.1111/bmsp.12028

[37] Leavitt K , Schabram K, Hariharan P et al Ghost in the machine: on organizational theory in the age of machine learning. Acad Manage Rev 2021;46:750–77.

[38] Chen J , Li X, Hallinger P et al Looking back and ahead: a bibliometric review of research on principal well-being, 1962–2022. Educ Manag Adm Leadersh 2023;

